# Changes in the Sodium Content of Foods Sold in Four Latin American Countries: 2015 to 2018

**DOI:** 10.3390/nu13114108

**Published:** 2021-11-16

**Authors:** Adriana Blanco-Metzler, Jaritza Vega-Solano, Beatriz Franco-Arellano, Lorena Allemandi, Rodrigo Burgos Larroza, Lorena Saavedra-Garcia, Madyson Weippert, Bridve Sivakumar, Karla Benavides-Aguilar, Victoria Tiscornia, Guillermo Sequera Buzarquis, Leila Guarnieri, Mayra Meza-Hernández, Felicia Cañete Villalba, Luciana Castronuovo, Alyssa Schermel, Mary R. L’Abbé, JoAnne Arcand

**Affiliations:** 1 Instituto Costarricense de Investigación y Enseñanza en Nutrición y Salud (INCIENSA), Apartado, Tres Rios 4-2250, Costa Rica; ablanco@inciensa.sa.cr (A.B.-M.); jarivega@gmail.com (J.V.-S.); kbenavides@inciensa.sa.cr (K.B.-A.); 2 Faculty of Health Sciences, Ontario Tech University, Oshawa, ON L1H 7k4, Canada; beatriz.francoarellano@ontariotechu.net (B.F.-A.); bridve.sivakumar@ontariotechu.ca (B.S.); 3 Department of Nutritional Sciences, Faculty of Medicine, University of Toronto, Toronto, ON M5S 1A1, Canada; madyson.weippert@mail.utoronto.ca (M.W.); a.schermel@gmail.com (A.S.); mary.labbe@utoronto.ca (M.R.L.); 4 Fundación Interamericana del Corazón, Arévalo 2364, Buenos Aires C1425, Argentina; lallemandi@gmail.com (L.A.); victoria.tiscornia@ficargentina.org (V.T.); leila.guarnieri@ficargentina.org (L.G.); luciana.castronuovo@ficargentina.org (L.C.); 5 Ministerio de Salud y Bienestar Social de Paraguay, Asunción 1001-1925, Paraguay; rburgos@mspbs.gov.py (R.B.L.); guillesequera@gmail.com (G.S.B.); feliciacanete@gmail.com (F.C.V.); 6 CRONICAS Center of Excellence in Chronic Diseases, Universidad Peruana Cayetano Heredia, Lima 15074, Peru; lorena.saavedrag@gmail.com (L.S.-G.); mayrameza24@gmail.com (M.M.-H.)

**Keywords:** sodium, sodium reduction, sodium targets, food supply, food policy, public health, global health

## Abstract

In 2015, the Pan American Health Organization (PAHO) published sodium targets for packaged foods, which included two distinct levels: one “regional” and one “lower” target. Changes to the sodium content of the food supply in Latin American Countries (LAC) has not been evaluated. A repeated cross-sectional study used food label data from 2015 (*n* = 3859) and 2018 (*n* = 5312) to determine changes in the proportion of packaged foods meeting the PAHO sodium targets and the distribution in the sodium content of foods in four LAC (Argentina, Costa Rica, Paraguay, Peru). Foods were classified into the 18 food categories in the PAHO targets. The proportion of foods meeting the regional targets increased from 82.9% to 89.3% between 2015 and 2018 (*p* < 0.001). Overall, 44.4% of categories had significant decreases in mean sodium content. Categories with a higher proportion of foods meeting the regional and lower targets in 2018 compared to 2015 (*p* < 0.05) were breaded meat and poultry, wet and dry soups, snacks, cakes, bread products, flavored cookies and crackers, and dry pasta and noodles. While positive progress has been made in reducing the sodium content of foods in LAC, sodium intakes in the region remain high. More stringent targets are required to support sodium reduction in LAC.

## 1. Introduction

Hypertension is a major modifiable risk factor for cardiovascular diseases (CVD), the leading cause of morbidity and mortality in Latin American Countries (LAC) [1,2]. In LAC, up to 40% of the adult population has hypertension, accounting for one in five deaths [2,3,4,5]. The prevention and management of hypertension is a priority for the World Health Organization (WHO) and Pan American Health Organization (PAHO). Reducing the prevalence of high blood pressure by 25% by 2025 is part of the WHO global action plan on noncommunicable diseases [6].

Excess sodium consumption increases blood pressure and subsequent risk of hypertension, CVD, stroke, and kidney disease [7,8]. Mean sodium intakes in most LAC exceeds the WHO recommended intake of 2000 mg sodium per day [9], with mean intakes often double the recommended levels [10,11,12]. In 2014, a global study estimated that the mean sodium intake is around 3200 mg/day in Latin America and the Caribbean, with adult males having higher intake compared to adult females [13]. For example, estimated mean daily sodium intake is 4480 mg in Argentina [14], 4600 mg in Costa Rica [15], 5480 mg in Paraguay [16], and 4400 mg in Peru [17]. A major source of dietary sodium is packaged foods. In Argentina, processed foods constitute 65–70% of the population’s sodium intake, with condiments, sauces, and spreads, as well as meat and meat products being among the food groups with the highest sodium content [14]. In Costa Rica, discretionary salt contributes to 60% of sodium intake in the diet, while 27% comes from packaged foods and condiments; however, over time there has been an increasing trend in the amount of sodium derived from packaged and prepared foods [15].

Recognizing the health burden associated with excess sodium intake, the WHO set a global target of reducing by 30% the mean population intake of salt by the year 2025 [6]. Public health strategies to address excess sodium typically focus on education, food labeling, and food reformulation [18]. To support LAC governments and food manufacturers in reducing the sodium content in packaged foods, in 2015 PAHO, with the advice of the Technical Advisory Group on Cardiovascular Disease Prevention through Population-wide Dietary Salt/Sodium Reduction, established a set of harmonized sodium targets for foods sold in the region. These voluntary harmonized sodium targets include 12 categories and a total of 18 subcategories for foods that contribute significant amounts of sodium to the diet (e.g., bread products) [19]. The targets include two levels: a “regional” target level and a “lower” target level [19]. These regional targets were endorsed by the SaltSmart Consortium in 2015, which comprised representatives from government, the food industry, and other key stakeholders in the region [19,20]. To date, there are few studies that have broadly assessed compliance with the regional sodium targets, or that have assessed changes in the sodium content, in packaged foods sold in LAC after the regional sodium targets were endorsed [21]. Therefore, the objective of this study was to assess the changes in the proportion of foods that met the regional sodium targets between 2015 and 2018 in Argentina, Costa Rica, Paraguay, and Peru. We also assessed the changes in the distribution of the sodium content of packaged foods that occurred between these two timepoints.

## 2. Materials and Methods

### 2.1. Study Design

This study was a repeated cross-sectional study using two datasets, containing nutrition information declared on the label of packaged foods, collected in 2015 and 2018. Data collection was guided by the 18 food subcategories in the regional sodium targets. Data were collected from four LAC countries (Argentina, Costa Rica, Paraguay, and Peru) using standardized methodologies. These countries were members of an International Development Research Centre (IDRC)-funded consortium that conducted research to inform dietary sodium reduction policies in LAC [22].

### 2.2. Sampling and Data Collection

In each country, food labels data were sampled from grocery store chains that had the greatest market share. Similar sampling and data acquisition methodologies were used in both 2015 and 2018. First, data from all packaged foods (except alcoholic beverages) were collected by systematically scanning grocery store shelves, capturing food label information through photographs [21]. If a product had more than one presentation (e.g., multiple package size), only one package size was collected as the nutrition information is the same per 100 g, regardless of the presentation. For the 2015 collection, digital cameras were used [23]. In 2018, the same information was collected using a mobile application called Food Labeling Information Program for Latin America (FLIP-LAC). The FLIP-LAC was an adapted version of FLIP© (created at the University of Toronto by M.R.L) that allowed for the integration of food label data for each of the participating LAC countries [24]. FLIP-LAC included a web and mobile application for food information data collection and processing, as described in detail elsewhere [25]. Data were collected between July 2015 and February 2016 for the first dataset, with most data collected in 2015 [21]. The second dataset was collected between September 2017 and December 2018, with most data collected in 2018. These two datasets are referred to as “2015” and “2018” in this article. Second, trained staff extracted nutrition information from food labels, which included product name, number of servings per package, serving size, and nutrient declarations (e.g., sodium, calories, sugars) per serving or per 100 g, or both. If salt was reported on the food label, it was converted to sodium equivalents considering a conversion factor of 23 mmol. For foods that required data to be reported “as prepared” or “as consumed” (cakes, pasta “as consumed”, noodles, wet and dry soups), recipes were created using the LATINFOODS database [26]. Data were captured in an Excel file for the 2015 collection and in the FLIP-LAC for the 2018 database. Finally, at least two researchers in each country independently classified foods collected in that country into one of the 18 food categories/subcategories that aligned with the PAHO targets (both “regional” target and “lower” target) [19], following a standardized protocol and validation process [27]. Any discrepancies in classification were discussed between the researchers. Food products that did not fall within one of the 18 food categories with a sodium target were excluded (e.g., candies and sweets, beverages). In 2015, products with only sodium declarations on the label were sampled. For 2018, all products on the shelves were sampled, but only products that included sodium declarations on the food label were included in the analysis. Quality assurance measures were implemented to detect duplicate products and ensure accuracy of data entry (e.g., rank ordering data to identify outliers, calculation of Atwater factors) and food category classification.

### 2.3. Statistical Analyses

The sodium content of packaged foods was expressed as a standardized unit of mg/100 g. The proportion of products in each food category meeting both regional target levels (regional target, lower target) were calculated for both data collection periods. Comparisons between the two timepoints were analyzed using a chi-square test, or Fisher’s exact test for cells with <5 counts. The mean, standard deviation, and distribution (minimum, maximum, and 25th, 50th [median], and 75th percentiles) of sodium content were calculated both overall and by food category. Statistically significant changes in the distribution of sodium between the two data collection periods were determined by the Kolmogorov–Smirnov test. A *p*-value < 0.05 was considered statistically significant. The variability in the sodium content of packaged foods available in the four LAC was estimated by the coefficient variability (SD/mean × 100). Categorical variables are presented as frequency (percent). Continuous variables are presented as the mean ± standard deviation. Analyses were conducted using IBM SPSS version 26.0 [28].

## 3. Results

This analysis included a total of 9171 foods, of which 3859 foods were from 2015 and 5312 foods were from 2018, from the four LAC countries and across 18 food categories (Table 1, Supplementary Table S1). In 2018, 92.6% of food labels collected provided information about sodium or salt (mg or g): 99.2% in Argentina, 82.1% in Costa Rica, 98.7% in Paraguay, and 88.8% in Peru (Supplementary Table S2).

### 3.1. Changes in the Proportion of Packaged Foods Meeting the Regional Target Levels

The proportion of packaged foods meeting the regional targets significantly increased from 82.9% to 89.3% between 2015 and 2018 (*p* < 0.001, Table 1). Seven food categories had a significantly higher proportion of foods meeting the regional targets from 2015 to 2018: breaded meat and poultry, from 61.1% to 83.3% (*p* < 0.001); wet and dry soups, from 62.7% to 81.1% (*p* < 0.001); snacks 76.0% to 85.7% (*p* < 0.001); cakes, from 63.1% to 78.9% (*p* < 0.001); bread products, from 78.0% to 93.2% (*p* < 0.001); flavored cookies and crackers, 96.1% to 99.2% (*p* = 0.041); and pasta and noodles, dry uncooked, 98.5% to 99.7% (*p* = 0.034). While some food categories had a slight decrease in the proportion of foods meeting the regional target, none achieved statistical significance. On examination of data in 2018, the greatest compliance with the regional target was among dry uncooked pasta and noodles (99.7%), while bouillon cubes and powders had the lowest level of compliance (57.7%).

### 3.2. Changes in the Proportion of Packaged Foods Meeting the Lower Sodium Target Levels

The overall proportion of foods meeting the lower sodium targets also significantly increased between 2015 and 2018 from 49.2% to 55.6% (*p* < 0.001, Table 1). The food categories with significantly greater proportions of foods meeting the lower target in 2018, compared to 2015, were breaded meat and poultry, from 16.7% to 54.6% (*p* < 0.001); wet and dry soups, from 40.6% to 60.1% (*p* < 0.001); cakes, from 24.7% to 44.7% (*p* < 0.001); flavored cookies and crackers, 55.6% to 67.9% (*p* < 0.009); bread products, from 35.1% to 46.6% (*p* < 0.001); pasta and noodles, dry uncooked, 93.5% to 99.1% (*p* < 0.001). On examination of data in 2018, dry uncooked pasta and noodles had the highest level of compliance with the lower regional target (99.1%), while mayonnaise had the lowest level of compliance (14.0%).

### 3.3. Changes in the Distribution of Sodium Levels between 2015 and 2018

On examination of the distribution of sodium levels between 2015 and 2018, 44.4% (8/18) of food categories overall had statistically significant decreases in sodium content, and 55.6% (10/18) of food categories had no statistically significant change in sodium content (Table 2). While some categories had higher levels of sodium in 2018, the change was not statistically significant. Food categories with statistically significant decreases in sodium (mean ± SD, mg/100 g) from 2015 to 2018 were pasta and noodles, dry uncooked (445 ± 628 to 84 ± 270; *p* < 0.001); breaded meat and poultry (756 ± 440 to 474 ± 272; *p* < 0.001); wet and dry soups (564 ± 899 to 298 ± 92; *p* < 0.001); cakes (374 ± 233 to 275 ± 218; *p* < 0.001); seasonings for side and main dishes (9475 ± 10739 to 5872± 7974, *p* = 0.010); bread products (446 ± 260 to 385 ± 192; *p* < 0.001); flavored cookies and crackers (651 ± 352 to 591 ± 275; *p* < 0.012); and snacks (715 ± 648 to 583 ± 353; *p* < 0.002) (Table 2). Median sodium levels showed similar trends. Seven categories had increases in sodium between 2015 and 2018, but these did not reach statistical significance, such as noodles in broth, cured and preserved meats, mayonnaise, bouillon cubes and powders, breakfast cereals, cookies and sweet cookies, and meat and sausages. At the individual country level, in 2018 some differences in the distribution and mean levels of sodium content of foods were observed (Supplementary Table S3). For example, the mean sodium content of breaded meat and poultry products was 352 ± 139 mg/100 g in Peru; however, the levels were twice as high in Costa Rica at 732 ± 240 mg/100 g.

In 2018, four categories exceeded 100% variability in sodium content: pasta and noodles, dry uncooked (321%), with the other four categories being cookies and sweet cookies (183%), seasonings for side and main dishes (136%), and cured and preserved meats (111.6%) (Table 2).

## 4. Discussion

To our knowledge, this is the most comprehensive analysis that has monitored the changes in the sodium content of packaged food in the Latin American region, including progress in meeting the 2015 PAHO regional sodium reduction targets. This study provides key data to inform public health nutrition policies in the region. It adds to the global literature monitoring the progress made in sodium reduction in low- and middle-income countries, in line with the WHO recommendations for achieving population-wide sodium reduction [29]. The timing of this analysis was ideal, being conducted at approximately the midpoint of the WHO’s global target of reducing salt consumption by 30% by 2025 [6]. a critical time point for countries to assess progress toward this target, and the need to update or revise policy and implementation strategies to achieve success.

In this study’s main findings, we observed statistically significant increases in the proportion of foods meeting the 2015 regional targets from 2015 to 2018: from 82.9% to 89.3% in foods meeting the regional target levels, and from 49.2% to 55.6% in foods meeting the lower target levels. When comparing these results country-level assessments in the LAC region and in other regions of the world, whether the targets were voluntary or mandatory, we found both similarities and differences. For example, similar to the current study, in Brazil [30] between 2011 to 2017, there was an 8–34% reduction in the mean sodium content in more than half of the categories, and a large proportion of foods (85%) met the PAHO regional targets in 2017 [30]. In the first evaluation of Canada’s voluntary sodium reduction targets (2010 to 2013), which used a similar collection approach as in this study, there was a slight increase in the proportion of foods that met at least one of the targets, from 51.4% to 58.2%, with only 16% of food categories having significantly reduced sodium levels [31]. Outside of the PAHO region, in 2008 the European Union Framework for National Salt Initiatives set a common minimum European benchmark of a minimum 16% reduction in the sodium content of foods, across all food products [32], with some positive progress in sodium reduction [33]. In Hungary, between 2009 and 2018 the proportion of breads with high sodium content (defined as >1.5 g salt/100 g) decreased from 60% to approximately 25%. In Ireland, between 2003 and 2015, sodium reduction was 13% in white bread, 27% in whole meal bread, 29% in wholegrain bread, 27% in bacon, 11% in sausages, cooked 15% in ham, 38% in multigrain breakfast cereals, and 63% in cornflake-type breakfast cereal [33]. This progress is particularly promising since bread is also a large contributor to sodium intakes in many countries of the LAC region. In the current analysis, the proportion of bread products meeting the regional and lower targets significantly increased from 78.0% to 93.2% and 35.1% to 46.6%, respectively. However, progress varied across countries (Supplementary Table S3). Our data highlight the need to not only monitor at the regional level, but also at the national level to ensure country-specific progress is being made. Country-level monitoring is critical since our analysis also revealed high variability in the distribution of sodium across products in the same subcategory. This finding confirms that the development and reformulation of products to be lower in sodium is feasible. In addition, it is evident that (1) it is technically feasible to reduce the sodium content of the foods for the food industry in the LAC region, (2) progressive and continuous efforts to reduce sodium among all the sectors involved is required, and (3) the successes and challenges of these efforts should be monitored and reported.

In this study a very high proportion of products were already meeting the regional and lower targets, both in 2015 and 2018. Almost 90% of products met the regional targets in 2018. Since sodium intakes in LAC remain unacceptably high despite the positive “progress” observed in the current study, our study and others highlight the need for more progressive regional targets to impact sodium intakes and improve CVD outcomes more effectively. Comparatively, the 2012 and 2020 voluntary sodium reduction targets developed by Health Canada are more stringent than the regional targets levels. However, a recent analysis showed that even if all Canadian packaged foods met the targets, they are not low enough to achieve sodium reduction goals [34]. Another earlier analysis across 14 Latin American and Caribbean countries revealed that 82% of foods met the regional target levels and 47% met the lower target levels [21]. Given the high levels of “success”, the authors called for more stringent harmonized regional sodium targets to better impact population-wide sodium reduction. The 2015 regional targets were set based on existing national targets in the region, an appropriate approach at the time since minimal data were available on the sodium content of LAC foods. In 2015, PAHO and the Salt Smart Consortium stated that the sodium reduction targets would be re-evaluated every two years, in 2016 and 2018 [19], which did not occur. In May 2021, the WHO global sodium benchmarks were released which included sodium targets for more than 60 categories of processed foods [35], taking a similar approach to the 2015 regional targets, which considered national-level sodium targets that had been adopted globally. While the WHO sodium benchmarks [35] can serve as a guide, it is important to adapt these targets to reflect the diverse array of cultural foods, recipes, and manufacturing practices in LAC and other countries in the PAHO region. Additionally, the food supply and food systems within a region are often interconnected. However, there are no known empirical data that suggest the extent to which the four countries in this study share a food supply. Despite this, it can be assumed that some multinational brands may distribute the same products (with the same formulation) to various countries. However, products of the same name and brand are also known have varying nutritional composition between countries [36], likely because of varying differences in the composition of the ingredients and/or taste preferences between countries and regions. This should be a topic for future analyses since reformulation efforts of multinational brands may have profound impacts on population sodium intakes. Finally, several companies in the LAC, including multinational companies, had committed to sodium reduction prior to 2015 in relevant products and brands [37,38]. These efforts were likely stimulated by international efforts to reduce dietary sodium. It is unknown if progress in reducing the sodium content of food was made prior to 2015, since few monitoring analyses were conducted before that time.

To further accentuate the importance of regional targets, this study also found meaningful increases in the number of packaged foods sold in the LAC from 2015 to 2018. Eleven categories had particularly strong growth over this timeframe, and unfortunately four of the eleven subcategories had increases in the sodium content over time. This growth reflects the global and regional trends of increasing availability and sales of ultra-processed foods [39]. Having a set of revised harmonized regional targets that are more progressive will potentially influence the development of new lower sodium products by food manufacturers that will support public health goals related to sodium.

Recently, the PAHO conducted a policy mapping survey of member countries. Many countries in the region have established policies related to sodium reduction, including setting sodium reduction targets for foods contributing the most dietary sodium: 11/34 countries have policies related to voluntary formulation (several of which include their own national targets), and 2/34 countries (Argentina and Paraguay) have established mandatory sodium reduction targets for key food categories [40]. However, only 9/34 countries have monitoring programs for the sodium content of foods [40]. Using the database generated from this study, Argentina [23,41] and Costa Rica [42] have also assessed compliance with their national sodium targets and have found 90% and 87% compliance, respectively. Data from this study can be used to inform policies related to sodium. Of the countries that participated in the current study, Argentina has developed national legislation for mandatory targets and has assessed compliance at numerous timepoints [23,41]. Likewise, Costa Rica evaluated compliance with their national voluntary targets [42] and used the data to update and expand their national targets (personal communication, A.B.-M.). Peru does not currently have national sodium targets but has a new front-of-pack labeling legislation, which includes warnings for products high in sodium [43]. Paraguay currently has a sodium target for artisanal breads; however, data from this study are being used to establish a mandatory target for processed breads (personal communication, R.B.L.). These policy actions illustrate the benefits of generating this type of data for policy advancements in the region. These analyses that inform public health policy on sodium reduction were possible with the support of the IDRC funded project “Scaling-up and evaluating policies and programs in salt reduction in LAC” [22]. This funding allowed for international collaborations that promoted capacity building, the development of technical research skills, and technological adaptations in the four low- and middle-income LAC in this study, which resulted in country-level success in research implementation and policy development [44].

This study has limitations that are important to discuss. The different sample sizes included between 2015 and 2018 or collecting food label data using electronic collection tools may highlight sampling variations that could impact the study results; however, similar sampling procedures and analysis methodologies were applied in each country and at both timepoints. The variance in the number of products included is likely due to the increasing availability of packaged foods in LAC, as described above. This study also relied on information presented on the Nutrition Facts table. However, a Costa Rican study showed that a very small proportion of food labels did not comply with the 20% variance that is permitted when reporting nutrient values on the food label [42]. Additionally, not all foods may report sodium information on the food label. This study showed most of foods (82.1% to 99.2%) had the sodium content declared on the package in 2018 (data from 2015 are not available), with nearly complete compliance in Paraguay and Argentina, where food labeling is mandatory. Finally, this study did not conduct analyses using sales-weighted data. Such data are costly and were not accessible for this study. However, since the regional sodium targets were developed considering national targets that did not use sales weighted data, an analysis using sales weighted data is not essential. Future research would also benefit from expanding the number of food categories so as to collect data for all foods that contribute sodium to the diet (e.g., cheese, mashed beans, corn tortillas) or for subcategories (e.g., white bread, whole grain bread, artisanal bread) to enable broadening the number of foods and subcategories with sodium reduction targets; however, the intention of this study was to examine progress in reformulation relative to foods that currently have a regional target level.

## 5. Conclusions

The proportion of packaged foods meeting the 2015 regional targets significantly increased from 82.9% to 89.3% for 2015 and 2018. Thirty-nine percent (7/18) food categories had a higher proportion of foods meeting the regional sodium targets, over time. Since a high proportion of foods were already meeting the targets at baseline, and sodium intakes in LAC remain unacceptably high, more stringent sodium targets should be promoted in the region to support further sodium reductions in packaged foods, and subsequently sodium intakes, in LAC. Planned, periodic food supply monitoring and evaluation is fundamental for countries to ensure they align sodium reduction policies and programs to achieve the WHO’s global target of 30% reduction in population sodium intakes by 2025.

## Figures and Tables

**Table 1:** Changes in the proportion of foods meeting the regional and lower targets in Argentina, Costa Rica, Paraguay, and Peru between 2015–2016 and 2017–2018.

Food Categories ^1^	Collection Year	Total ^2^	Meeting Regional Targets ^3^			Meeting Lower Targets ^3^		
		*n*	*n*	*%*	*p*	*n*	*%*	*p*
Bread products	2015	350	273	78.0	<0.001	123	35.1	0.003
	2018	309	288	93.2	-	144	46.6	-
Breakfast cereals	2015	392	350	89.3	0.175	309	78.8	0.138
	2018	427	393	92.0	-	354	82.9	-
Butter and margarine	2015	101	82	81.2	0.962	52	51.5	0.320
	2018	140	114	81.4	-	63	45.0	-
Cakes	2015	312	197	63.1	<0.001	77	24.7	<0.001
	2018	237	187	78.9	-	106	44.7	-
Bouillon cubes and powders	2015	79	43	54.4	0.681	23	29.1	0.293
	2018	78	45	57.7	-	17	21.8	-
Meat and fish seasonings	2015	58	52	89.7	0.963	52	89.7	0.963
	2018	85	76	89.4	-	76	89.4	-
Seasonings for side and main dishes	2015	145	141	97.2	0.303	91	62.8	0.054
	2018	74	74	100.0	-	56	75.7	-
Cookies and sweet cookies	2015	432	408	94..4	0.355	222	51.4	0.094
	2018	930	889	95.6	-	523	56.2	-
Flavored cookies and crackers	2015	180	173	96.1	0.041	100	55.6	0.009
	2018	243	241	99.2	-	165	67.9	-
Mayonnaise	2015	90	86	95.6	0.767	21	23.3	0.071
	2018	136	128	94.1	-	19	14.0	-
Meats and sausages	2015	378	328	86.8	0.312	118	31.2	0.351
	2018	498	420	84.3	-	141	28.3	-
Cured and preserved meats	2015	41	33	80.5	0.695	19	46.3	0.907
	2018	84	70	83.3	-	38	45.2	-
Breaded meat and poultry	2015	72	44	61.1	0.001	12	16.7	<0.001
	2018	108	90	83.3	-	59	54.6	-
Pasta and noodles, as consumed	2015	n/a	n/a	n/a	n/a	n/a	n/a	n/a
	2018	263	182	69.2	-	84	31.9	-
Pasta and noodles, dry uncooked	2015	337	332	98.5	0.034	315	93.5	<0.001
	2018	738	736	99.7	-	731	99.1	-
Snacks	2015	604	459	76.0	<0.001	237	39.2	0.423
	2018	749	642	85.7	-	310	41.4	-
Noodles in broth	2015	71	61	85.9	0.119	38	53.5	0.617
	2018	65	49	75.4	-	32	49.2	-
Wet and dry soups	2015	217	136	62.7	<0.001	88	40.6	<0.001
	2018	148	120	81.1	-	89	60.1	-
TOTAL 2015		3859	3198	82.9	<0.001	1897	49.2	<0.001
TOTAL 2018		5312	4744	89.3	-	3007	56.6	-

^1^ Food categories as defined by Pan American Health Organization/SALT-SMART Consortium consensus statement to advance target harmonization by agreeing on regional targets for the salt/sodium content of key food categories (2015) [19]. There are two PAHO regional target levels for each food category: The regional target level which serves is a maximum, and a lower target level. ^2^ Includes only products with declared sodium content on the food label. ^3^ Statistically significant changes in the proportions meeting the regional (maximum) and the lower sodium targets were determined by the chi-square test or Fisher’s exact test for cells with <5 counts. A *p* value < 0.05 was considered statistically significant.

**Table 2:** Changes in the sodium content of packaged foods (mg per 100 g) in Argentina, Costa Rica, Paraguay, and Peru between 2015–2016 and 2017–2019 (*n* = 9171).

Food Categories ^1^		All	Products with Sodium Data ^2^		Sodium (mg per 100g/mL)			Percentiles (mg per 100g/mL)					
	Year	*n*	*n*	*%*	Mean	SD	Min	25th	50th	75th	Max	Mean Δ	*p* ^3^
Bread products	2015	350	350	100.0	446	260	0	306	475	580	1300	0.228	<0.001
	2018	353	309	87.5	385	192	0	316	432	500	1030	-	-
Breakfast cereals	2015	392	392	100.0	307	247	0	100	283	468	1395	0.084	0.114
	2018	432	427	98.8	318	262	0	137	290	437	2700	-	-
Butter and margarine	2015	101	101	100.0	554	652	0	140	480	670	5000	0.068	0.951
	2018	145	140	96.6	486	347	0	140	571	707	1571	-	-
Cakes	2015	312	312	100.0	374	233	0	212	310	508	1465	0.248	<0.01
	2018	334	237	71.0	275	218	0	129	230	371	1739	-	-
Bouillon cubes and powders	2015	79	79	100.0	19,018	7356	263	16,667	20,000	23,120	33,810	0.098	0.845
	2018	83	78	94.0	19,579	6801	139	18,800	19,880	22,300	33,813	-	-
Meat and fish seasonings	2015	58	58	100.0	12,130	9834	17	3900	11,371	19,710	38,000	0.117	0.728
	2018	90	85	94.4	11,209	8892	4	3667	10,146	16,340	36,140	-	-
Seasonings for side and main dishes	2015	145	145	100.0	9475	10,739	0	767	3889	18,200	37,280	0.233	0.010
	2018	77	74	96.1	5872	7974	0	400	1067	8444	28,000	-	-
Cookies and sweet cookies	2015	432	432	10.0	283	223	0	177	260	343	3433	0.079	0.050
	2018	976	930	95.3	289	529	0	177	240	313	9000	-	-
Flavored cookies and crackers	2015	180	180	100.0	651	352	0	498	657	804	1929	0.157	0.012
	2018	243	243	100.0	591	275	0	473	594	759	2000	-	-
Mayonnaise	2015	90	90	100.0	814	230	96	700	850	942	2000	0.140	0.242
	2018	139	136	97.8	897	630	1	767	850	942	7698	-	-
Meats and sausages	2015	378	378	100.0	865	587	0	640	818	1030	7000	0.069	0.260
	2018	565	498	88.1	890	412	2	658	834	1076	2720	-	-
Cured and preserved meats	2015	41	41	100.0	1433	962	0	596	1540	1700	3500	0.148	0.587
	2018	142	84	59.2	1580	1761	153	822	1403	1764	15,400	-	-
Breaded meat and poultry	2015	72	72	100.0	756	440	71	511	617	900	2110	0.426	<0.001
	2018	134	108	80.6	474	272	1	284	446	620	1139	-	-
Pasta and noodles, as consumed	2015	n/a	n/a	n/a	n/a	n/a	n/a	n/a	n/a	n/a	n/a	n/a	n/a
	2018	271	263	97.0	517	288	0	340	553	664	2496	-	-
Pasta and noodles, dry uncooked	2015	337	337	100,0	445	628	0	10	190	735	7000	0.446	<0.001
	2018	770	738	95.8	84	270	0	0	7	16	2283	-	-
Snacks	2015	604	604	100.0	715	648	0	399	609	891	8000	0.104	0.002
	2018	765	749	97.9	583	353	0	361	596	760	2667	-	-
Noodles in broth	2015	71	71	100.0	349	92	108	304	345	396	640	0.162	0.336
	2018	65	65	100.0	381	114	156	308	365	424	900	-	-
Wet and dry soups	2015	217	217	100.0	564	899	2	260	322	621	5900	0.285	<0.001
	2018	151	148	98.0	298	92	40	255	299	330	725	-	-
TOTAL 2015		3859	3859	100.0	1428	4281	0	239	470	800	38,000	0.102	<0.001
TOTAL 2018		5735	5312	92.6	973	3226	0	151	367	671	36,140	-	-

^1^ Food categories as defined by Pan American Health Organization/SALT-SMART Consortium consensus statement to advance target harmonization by agreeing on regional targets for the salt/sodium content of key food categories (2015), available at: https://www.paho.org/hq/dmdocuments/2015/salt-smart-Consensus-statement-with-targets-FINAL.pdf, accessed on 10 November 2021. ^2^ Sodium content was recorded “as consumed”. ^3^ Statistically significant changes in the distribution of sodium were determined by the Kolmogorov–Smirnov test. A *p* value < 0.05 was considered statistically significant.

## Data Availability

Adapted from https://idl-bnc-idrc.dspacedirect.org/handle/10625/58989 accessed on 10 November 2021.

## Supplementary Materials

The following are available online at https://www.mdpi.com/article/10.3390/nu13114108/s1, Table S1: Number of packaged foods per food category by country included in the analysis (*n* = 9171), Table S2: Number and proportion of food labels per food category with sodium data in the 2018 database, Table S3: Distribution of sodium content of packaged foods (mg/100 g) in 2018, by food category and country.

## References

[1] Ordunez P., Martinez R., Niebylski M.L., Campbell N.R. (2015). Hypertension prevention and control in Latin America and the Caribbean. J. Clin. Hypertens. (Greenwich).

[2] Kearney P.M., Whelton M., Reynolds K., Muntner P., Whelton P.K., He J. (2005). Global burden of hypertension: Analysis of worldwide data. Lancet.

[3] Ruilope L.M., Chagas A.C., Brandao A.A., Gomez-Berroteran R., Alcala J.J., Paris J.V., Cerda J.J. (2017). Hypertension in Latin America: Current perspectives on trends and characteristics. Hipertens Riesgo Vasc..

[4] Sanchez R.A., Ayala M., Baglivo H., Velazquez C., Burlando G., Kohlmann O., Jimenez J., Jaramillo P.L., Brandao A., Valdes G. (2009). Latin American guidelines on hypertension. Latin American Expert Group. J. Hypertens..

[5] NCD Risk Factor Collaboration (NCD-RisC) (2021). Worldwide trends in hypertension prevalence and progress in treatment and control from 1990 to 2019: A pooled analysis of 1201 population-representative studies with 104 million participants. Lancet.

[6] World Health Organization Global Action Plan for the Prevention and Control of Noncommunicable Diseases 2013–2020. http://apps.who.int/iris/bitstream/10665/94384/1/9789241506236_eng.pdf?ua=1.

[7] Strazzullo P., D’Elia L., Kandala N.B., Cappuccio F.P. (2009). Salt intake, stroke, and cardiovascular disease: Meta-analysis of prospective studies. BMJ.

[8] Aburto N.J., Ziolkovska A., Hooper L., Elliott P., Cappuccio F.P., Meerpohl J.J. (2013). Effect of lower sodium intake on health: Systematic review and meta-analyses. BMJ.

[9] World Health Organization Diet, Nutrition and the Prevention of Chronic Diseases. http://apps.who.int/iris/bitstream/10665/42665/1/WHO_TRS_916.pdf.

[10] Lamelas P., Diaz R., Orlandini A., Avezum A., Oliveira G., Mattos A., Lanas F., Seron P., Oliveros M.J., Lopez-Jaramillo P. (2019). Prevalence, awareness, treatment and control of hypertension in rural and urban communities in Latin American Countries. J. Hypertens..

[11] Brown I.J., Tzoulaki I., Candeias V., Elliott P. (2009). Salt intakes around the world: Implications for public health. Int. J. Epidemiol..

[12] Pesantes M.A., Diez-Canseco F., Bernabé-Ortiz A., Ponce-Lucero V., Miranda J.J. (2017). Taste, salt consumption, and local explanations around hypertension in a rural population in northern Peru. Nutrients.

[13] Mozaffarian D., Fahimi S., Singh G.M., Micha R., Khatibzadeh S., Engell R.E., Lim S., Danaei G., Ezzati M., Powles J. (2014). Global sodium consumption and death from cardiovascular causes. N. Engl. J. Med..

[14] Ferrante D., Apro N., Ferreira V., Virgolini M., Aguilar V., Sosa M., Perel P., Casas J. (2011). Feasibility of salt reduction in processed foods in Argentina. Rev. Panam. Salud. Publica.

[15] Blanco-Metzler A., Moreira Claro R., Heredia-Blonval K., Caravaca Rodríguez I., Montero-Campos M.L.A., Legetic B., L’Abbe M.R. (2017). Baseline and estimated trends of sodium availability and food sources in the Costa Rican population during 2004–2005 and 2012–2013. Nutrients.

[16] Sequera V., Cañete F., Paiva T., Giménez E., Santacruz E., Fretes G., Benítez G. (2017). Patrones de excreción urinaria de sodio en población adulta en muestras de orina espontánea. Anales de la Facultad de Ciencias Médicas (Asunción).

[17] Carrillo-Larco R.M., Saavedra-Garcia L., Miranda J.J., Sacksteder K.A., Diez-Canseco F., Gilman R.H., Bernabe-Ortiz A. (2018). Sodium and potassium consumption in a semi-urban area in Peru: Evaluation of a population-based 24-hour urine collection. Nutrients.

[18] Santos J.A., Tekle D., Rosewarne E., Flexner N., Cobb L., Al-Jawaldeh A., Kim W.J., Breda J., Whiting S., Campbell N. (2021). A systematic review of salt reduction initiatives around the world: A midterm evaluation of progress towards the 2025 global non-communicable diseases salt reduction target. Adv. Nutr..

[19] Pan American Health Organization Saltsmart Consortium Consensus Statement to Advance Target Harmonization by Agreeing on Regional Targets for the Salt/Sodium Content of Key Food Categories. https://www.paho.org/hq/dmdocuments/2015/salt-smart-Consensus-statement-with-targets-FINAL.pdf.

[20] Campbell N., Legowski B., Legetic B., Ferrante D., Nilson E., Campbell C., L’Abbé M. (2014). Targets and timelines for reducing salt in processed food in the Americas. J. Clin. Hypertens. (Greenwich).

[21] Arcand J., Blanco-Metzler A., Benavides Aguilar K., L’Abbe M.R., Legetic B. (2019). Sodium levels in packaged foods sold in 14 Latin American and Caribbean Countries: A food label analysis. Nutrients.

[22] International Development Research Centre Scaling up and Evaluating Salt Reduction Policies and Programs in Latin American Countries. https://www.idrc.ca/en/project/scaling-and-evaluating-salt-reduction-policies-and-programs-latin-american-countries.

[23] Allemandi L., Tiscornia M.V., Ponce M., Castronuovo L., Dunford E., Schoj V. (2015). Sodium content in processed foods in Argentina: Compliance with the national law. Cardiovasc. Diagn Ther..

[24] Blanco-Metzler A., L’Abbé M., Arcand J., Montero M.A., Allemandi L., Nilson E., Cañete F., Saavedra L., Khaliq Pasha M., Ramos E. Final technical report/rapport technique. Scaling up and Evaluating Salt Reduction Policies and Programs in Latin American Countries. https://idl-bnc-idrc.dspacedirect.org/bitstream/handle/10625/58988/59109.pdf?sequence=1&isAllowed=y.

[25] Bernstein J.T., Schermel A., Mills C.M., L’Abbe M.R. (2016). Total and free sugar content of canadian prepackaged foods and beverages. Nutrients.

[26] Red Latinoamericana de Composición de Alimentos Tabla LATINFOODS. http://latinfoods.inta.cl/592-2/.

[27] Schermel A., Vega J., Franco-Arellano B., Arcand J.A., L’Abbe M.R., Blanco-Metzler A. Protocol for Flip Study of Project Idrc 180167 Scaling-up and Evaluating Salt Reduction Policies and Programs in Latin American Countries. https://idl-bnc-idrc.dspacedirect.org/bitstream/handle/10625/58973/59092.pdf.

[28] R Foundation R: A Language and Environment for Statistical Computing. https://www.R-project.org/.

[29] World Health Organization The Shake Technical Package for Salt Reduction. http://www.who.int/dietphysicalactivity/publications/shake-salt-habit/en/.

[30] Nilson E.A.F., Spaniol A.M., Gonçalves V.S.S., Moura I., Silva S.A., L’Abbé M., Jaime P.C. (2017). Sodium reduction in processed foods in Brazil: Analysis of food categories and voluntary targets from 2011 to 2017. Nutrients.

[31] Arcand J., Jefferson K., Schermel A., Shah F., Trang S., Kutlesa D., Lou W., L’Abbe M.R. (2016). Examination of food industry progress in reducing the sodium content of packaged foods in Canada: 2010 to 2013. Appl. Physiol. Nutr. Metab..

[32] The High Level Group on Diet P.A.a.H. National Salt Initiatives Implementing the eu Framework for Salt Reduction Initiatives. https://ec.europa.eu/health/ph_determinants/life_style/nutrition/documents/national_salt_en.pdf.

[33] World Health Organization Regional Office for Europe 11th Meeting of the Who Action Network on Salt Reduction in the Population in the European Region (esan). https://www.euro.who.int/__data/assets/pdf_file/0019/426205/01.29-ESAN-meeting-Bern-May-2019-report.pdf.

[34] Smith B.T., Hack S., Jessri M., Arcand J., McLaren L., L’Abbé M.R., Anderson L.N., Hobin E., Hammond D., Manson H. (2021). The equity and effectiveness of achieving Canada’s voluntary sodium reduction guidance targets: A modelling study using the 2015 Canadian community health survey—Nutrition. Nutrients.

[35] World Health Organization Global Sodium Targets. https://www.who.int/publications/i/item/9789240025097.

[36] He F.J., Jenner K.H., Macgregor G.A. (2010). Wash-world action on salt and health. Kidney Int..

[37] Nestlé Creación de Valor Compartido y Cumplimiento de Nuestros Compromisos. https://www.nestle.com/sites/default/files/asset-library/documents/library/documents/corporate_social_responsibility/nestle-csv-summary-report-2012-sp.pdf.

[38] El Comercio (2010). Pepsico reducirá Sodio, Azúcar y Grasas en sus Productos; Empresa Editora El Comercio Jr. Santa Rosa #300 Lima 1 Perú..

[39] Pan American Health Organization Ultra-Processed Food and Drink Products in Latin America: Trends, Impact on Obesity, Policy Implications. https://iris.paho.org/bitstream/handle/10665.2/7699/9789275118641_eng.pdf.

[40] Pan American Health Organization Salt/Sodium Intake Reduction Policies. https://www.paho.org/en/noncommunicable-diseases-and-mental-health/noncommunicable-diseases-and-mental-health-data-29.

[41] Allemandi L., Tiscornia M.V., Guarnieri L., Castronuovo L., Martins E. (2019). Monitoring sodium content in processed foods in Argentina 2017-2018: Compliance with national legislation and regional targets. Nutrients.

[42] Vega-Solano J., Blanco-Metzler A., Benavides-Aguilar K.F., Arcand J. (2019). An evaluation of the sodium content and compliance with the national sodium reduction targets among packaged foods sold in Costa Rica in 2015 and 2018. Nutrients.

[43] Meza-Hernández M., Villarreal-Zegarra D., Saavedra-Garcia L. (2020). Nutritional quality of food and beverages offered in supermarkets of Lima according to the peruvian law of healthy eating. Nutrients.

[44] Arcand J., Mosley J. Final Technical Report. Anexe iv-b) Program Evaluation. https://idl-bnc-idrc.dspacedirect.org/bitstream/handle/10625/58972/59107.pdf.

