# Supplementary material for: Changes in the Sodium Content of Foods Sold in Four Latin American Countries: 2015 to 2018
Source: Nutrients. 2021 Nov 16;13(11):4108. doi: 10.3390/nu13114108 (PMC8624930; doi:10.3390/nu13114108)
Supplement: Supplementary file 1 [file nutrients-13-04108-s001.zip › nutrients-1444404-supplementary.pdf]

Table S1. Number of packaged foods per food category by country included in the analysis (*n*=9,171).

**PAHO categories**[1]	**Collection *year***	**All countries *n***	**Argentina *n***	**Costa Rica *n***	**Paraguay *n***	**Peru *n***
Bread products	2015	350	123	83	76	68
	2018	309	110	85	91	23
Breakfast cereals	2015	392	88	115	98	91
	2018	427	85	135	128	79
Butter and margarine	2015	101	23	43	17	18
	2018	140	38	44	40	18
Cakes	2015	312	127	117	35	33
	2018	237	n/a	102	135	n/a
Bouillon cubes and powders	2015	79	23	26	6	24
	2018	78	38	13	27	n/a
Meat and fish seasonings	2015	58	26	15	6	11
	2018	85	19	45	21	n/a
Seasonings for side and main dishes	2015	145	22	44	28	51
	2018	74	26	8	40	n/a
Cookies and sweet cookies	2015	432	98	127	116	91
	2018	930	235	286	381	28
Flavored cookies and crackers	2015	180	67	43	32	38
	2018	243	76	81	85	1
Mayonnaise	2015	90	18	34	18	20
	2018	136	35	32	56	13
Meats and sausages	2015	378	162	60	109	47
	2018	498	207	107	177	7
Cured and preserved meats	2015	41	28	n/a	12	1
	2018	84	14	48	22	n/a
Breaded meat and poultry	2015	72	22	41	8	1
	2018	108	10	20	40	38
Pasta and noodles, as consumed	2015	n/a	n/a	n/a	n/a	n/a
	2018	263	129	7	127	n/a
Pasta and noodles, dry uncooked	2015	337	96	48	120	73
	2018	738	138	179	419	2
Snacks	2015	604	123	206	159	116
	2018	749	165	347	237	n/a
Noodles in broth	2015	71	n/a	13	3	55
	2018	65	9	13	23	20
Wet and dry soups	2015	217	79	71	28	39
	2018	148	49	77	22	n/a
**TOTAL 2015**	2015	**3859**	**1125**	**1086**	**871**	**777**
**TOTAL 2018**	2018	**5312**	**1383**	**1629**	**2071**	**229**

[1] Food categories as defined by Pan American Health Organization. SALT-SMART Consortium consensus statement to advance target harmonization by agreeing on regional targets for the salt/sodium content of key food categories. 2015; https://www.paho.org/hq/dmdocuments/2015/salt-smart-Consensus-statement-with-targets-FINAL.pdf, accessed on 12 November 2021.

Table S2. Number and proportion of food labels per food category with sodium data in the 2018 database

**Food categories**[1]	**Collection**	**Argentina**			**Costa Rica**			**Paraguay**			**Peru**		
		**All**	**Products with sodium data**		**All**	**Products with sodium data**		**All**	**Products with sodium data**		**All**	**Products with sodium data**	
	year	*n*	*n*[2]	*%*[2]	*n*	*n*	*%*	*n*	*n*	*%*	*n*	*n*	*%*
Bread products	2015	123	123		83	83	100.0	76	76	100.0	68	68	100.0
	2018	111	110	99.1	124	85	68.5	95	91	95.8	23	23	100.0
Breakfast cereals	2015	88	88		115	115	100.0	98	98	100.0	91	91	100.0
	2018	85	85	100.0	135	135	100.0	130	128	98.5	82	79	96.3
Butter and margarine	2015	23	23		43	43	100.0	17	17	100.0	18	18	100.0
	2018	38	38	100.0	49	44	89.8	40	40	100.0	18	18	100.0
Cakes	2015	127	127		117	117	100.0	35	35	100.0	33	33	100.0
	2018	n/a	n/a	n/a	194	102	52.6	140	135	96.4	n/a	n/a	n/a
Bouillon cubes and powders	2015	23	23		26	26	100.0	6	6	100.0	24	24	100,0
	2018	38	38	100.0	18	13	72.2	27	27	100.0	n/a	n/a	n/a
Meat and fish seasonings	2015	26	26		15	15	100.0	6	6	100.0	11	11	100.0
	2018	19	19	100.0	50	45	90.0	21	21	100.0	n/a	n/a	n/a
Seasonings for side and main dishes	2015	22	22		44	44	100.0	28	28	100.0	51	51	100.0
	2018	27	26	96.3	9	8	88.9	41	40	976	n/a	n/a	n/a
Cookies and sweet cookies	2015	98	98		127	127	100.0	116	116	100.0	91	91	100.0
	2018	237	235	99.2	326	286	87.7	382	381	99.7	31	28	90,3
Flavored cookies and crackers	2015	67	67		43	43	100.0	32	32	100.0	38	38	100.0
	2018	76	76	100.0	81	81	100.0	85	85	100.0	1	1	100.0
Mayonnaise	2015	18	18		34	34	100.0	18	18	100.0	20	20	100.0
	2018	35	35	100.0	32	32	100.0	56	56	100.0	16	13	81.3
Meats and sausages	2015	162	162		60	60	100.0	109	109	100.0	47	47	100.0
	2018	207	207	100.0	169	107	63.3	182	177	97.3	7	7	100.0
Cured and preserved meats	2015	28	28		n/a	n/a	n/a	12	12	100.0	1	1	100.0

**Food categories**[1]	**Collection**	**Argentina**			**Costa Rica**			**Paraguay**			**Peru**		
		**All**	**Products with sodium data**		**All**	**Products with sodium data**		**All**	**Products with sodium data**		**All**	**Products with sodium data**	
	year	*n*	*n*[2]	*%*[2]	*n*	*n*	*%*	*n*	*n*	*%*	*n*	*n*	*%*
	2018	14	14	100.0	105	48	45.7	23	22	95.7	n/a	n/a	n/a
Breaded meat and poultry	2015	22	22		41	41	100.0	8	8	100.0	1	1	100.0
	2018	10	10	100.0	26	20	76.9	41	40	97.6	57	38	66.7
Pasta and noodles, as consumed	2015	n/a	n/a		n/a	n/a	n/a	n/a	n/a	n/a	n/a	n/a	n/a
	2018	134	129	96.3	9	7	77.8	128	127	99.2	n/a	n/a	n/a
Pasta and noodles, dry uncooked	2015	96	96		48	48	100.0	120	120	100.0	73	73	100.0
	2018	140	138	98.6	203	179	88.2	424	419	98.8	3	2	66.7
Snacks	2015	123	123		206	206	100,0	159	159	100.0	116	116	100.0
	2018	165	165	100.0	361	347	96.1	239	237	99.2	n/a	n/a	n/a
Noodles in broth	2015	n/a	n/a		13	13	100.0	3	3	100.0	55	55	100.0
	2018	9	9	100.0	13	13	100.0	23	23	100.0	20	20	100.0
Wet and dry soups	2015	79	79		71	71	100.0	28	28	100.0	39	39	100.0
	2018	49	49	100.0	80	77	96.3	22	22	100.0	n/a	n/a	n/a
**TOTAL 2015-2016**		1125	1125	100.0	1086	1086	100.0	871	871	100.0	777	777	100.0
**TOTAL 2017-2018**		1394	1383	99.2	1984	1629	82.1	2099	2071	98.7	258	229	88.8

[1] Food categories as defined by Pan American Health Organization. SALT-SMART Consortium consensus statement to advance target harmonization by agreeing on regional targets for the salt/sodium content of key food categories. 2015; https://www.paho.org/hq/dmdocuments/2015/salt-smart-Consensus-statement-with-targets-FINAL.pdf, accessed on 12 November 2021.
[2] In 2015, only foods that contained sodium information on the food label were collected, therefore 100% of products carried sodium information

Commented [M1]: There is no explanation for “2” in the table.

Table S3. Distribution of sodium content of packaged foods (mg/100g) in 2018, by food category and country

**Food categories**[1]	**Country**	**Products with sodium data**	**Sodium (mg per 100g/ml)**		**Percentiles (mg per 100g/ml)**				
		n	Mean	SD	Min	25th	50th	75th	Max
Bread products	Argentina	110	443	126	1	396	443	500	1030
	Costa Rica	85	368	228	0	197	402	500	947
	Paraguay	91	354	200	0	224	410	500	707
	Peru	23	291	215	1	1	359	440	623
Breakfast cereals	Argentina	85	283	196	0	130	250	417	810
	Costa Rica	135	410	277	0	246	371	517	2500
	Paraguay	128	232	277	0	74	199	315	2700
	Peru	79	338	222	0	159	337	430	955
Butter and margarine	Argentina	38	272	239	0	92	190	540	810
	Costa Rica	44	594	265	0	509	643	732	1067
	Paraguay	40	487	371	0	140	555	670	1110
	Peru	18	673	442	0	450	761	1000	1571
Cakes	Argentina	n/a	n/a	n/a	n/a	n/a	n/a	n/a	n/a
	Costa Rica	102	358	269	4	172	330	456	1739
	Paraguay	135	212	142	0	108	167	275	597
	Peru	n/a	n/a	n/a	n/a	n/a	n/a	n/a	n/a
Bouillon cubes and powders	Argentina	38	20482	7224	1778	18989	20745	24267	33813
	Costa Rica	13	17834	7858	1000	18800	20000	21200	25400
	Paraguay	27	19149	5596	139	18421	19760	20760	33120
	Peru	n/a	n/a	n/a	n/a	n/a	n/a	n/a	n/a

Table S3. Cont.

Food categories[1]	Country	Products with sodium data	Sodium (mg per 100g/ml)		Percentiles (mg per 100g/ml)				
		n	Mean	SD	Min	25th	50th	75th	Max
Seasonings for side and main dishes	Argentina	n/a	n/a	n/a	n/a	n/a	n/a	n/a	n/a
	Costa Rica	8	16269	9755	6000	8000	14622	25350	28000
	Paraguay	202	606	1949	1	235	400	476	20340
	Peru	n/a	n/a	n/a	n/a	n/a	n/a	n/a	n/a
Cookies and sweet cookies	Argentina	362	312	211	0	164	281	429	1120
	Costa Rica	284	274	242	0	179	254	320	3419
	Paraguay	419	276	189	0	177	243	320	1240
	Peru	113	343	209	0	217	283	433	1111
Flavored cookies and crackers	Argentina	25	852	304	0	643	859	1124	1237
	Costa Rica	84	710	227	5	563	719	864	1433
	Paraguay	24	627	361	0	520	600	664	2000
	Peru	2	700	141	600	650	700	750	800
Mayonnaise	Argentina	35	862	91	550	842	850	925	1042
	Costa Rica	32	1064	1262	487	680	767	895	7698
	Paraguay	56	877	126	333	850	925	942	1025
	Peru	12	665	350	1	472	617	1000	1133
Meats and sausages	Argentina	173	881	281	150	746	844	1045	2142
	Costa Rica	147	1135	1275	2	754	1000	1349	15400
	Paraguay	30	791	403	235	600	680	840	2098
	Peru	7	1026	684	301	310	1320	1618	1708
Cured and preserved meats	Argentina	40	1634	655	115	1373	1504	1740	3623
	Costa Rica	8	1173	961	273	536	573	2093	2714
	Paraguay	30	1045	555	295	658	904	1256	2423
	Peru	n/a	n/a	n/a	n/a	n/a	n/a	n/a	n/a
Breaded meat and poultry	Argentina	17	469	164	46	408	517	590	618
	Costa Rica	20	732	240	375	543	740	904	1139
	Paraguay	1	668	n/a	668	668	668	668	668
	Peru	39	340	159	1	241	360	446	630

Table S3. Cont.

Meat and fish seasonings	Argentina	19	14423	4309	7829	10167	15500	17100	23493
	Costa Rica	45	8284	9138	4	1333	5517	12500	33636
	Paraguay	21	14567	9525	20	9420	15640	18520	36140
	Peru	n/a	n/a	n/a	n/a	n/a	n/a	n/a	n/a
Seasonings for side and main dishes	Argentina	26	5935	6840	0	1008	3164	12611	21707
	Costa Rica	8	16269	9755	6000	7556	14622	25900	28000
	Paraguay	40	3752	6721	74	240	400	1656	20600
	Peru	n/a	n/a	n/a	n/a	n/a	n/a	n/a	n/a
Cookies and sweet cookies	Argentina	235	241	130	0	153	231	313	1120
	Costa Rica	286	269	242	0	168	252	320	3419
	Paraguay	381	336	790	0	187	240	310	9000
	Peru	28	270	174	0	200	232	352	714
Flavored cookies and crackers	Argentina	76	500	239	0	433	547	633	861
	Costa Rica	81	701	222	5	552	702	848	1433
	Paraguay	85	567	316	0	430	573	729	2000
	Peru	1	600		600	600	600	600	600
Mayonnaise	Argentina	35	862	91	550	842	850	925	1042
	Costa Rica	32	1064	1262	487	668	767	896	7698
	Paraguay	56	877	126	333	850	925	942	1025
	Peru	13	666	335	1	505	633	1000	1133
Meats and sausages	Argentina	207	940	370	46	746	850	1084	2200
	Costa Rica	107	932	459	2	701	929	1198	2321
	Paraguay	177	800	403	54	570	728	920	2720
	Peru	7	1026	684	301	304	1320	1650	1708
Cured and preserved meats	Argentina	14	2011	769	1380	1405	1763	2250	3623
	Costa Rica	48	1570	2105	273	822	1350	1717	15400
	Paraguay	22	1328	1339	153	471	1039	1658	6475
	Peru	n/a	n/a	n/a	n/a	n/a	n/a	n/a	n/a

Table S3. Cont.

Breaded meat and poultry	Argentina	10	524	209	152	398	498	590	843
	Costa Rica	20	732	240	375	531	740	908	1139
	Paraguay	40	449	311	54	119	452	668	1030
	Peru	38	352	139	1	269	357	446	630
Pasta and noodles, as consumed	Argentina	129	494	237	0	360	536	640	1075
	Costa Rica	7	325	70	250	283	300	354	462
	Paraguay	127	552	333	0	351	593	711	2496
	Peru	n/a	n/a	n/a	n/a	n/a	n/a	n/a	n/a
Pasta and noodles, dry uncooked	Argentina	138	46	139	0	7	10	17	916
	Costa Rica	179	124	312	0	0	0	14	2040
	Paraguay	419	80	283	0	0	10	20	2283
	Peru	2	1	1	0	0	1	1	1
Snacks	Argentina	165	634	231	0	500	608	752	1237
	Costa Rica	347	580	410	0	272	536	780	2667
	Paraguay	237	552	328	0	420	600	720	1237
	Peru	n/a	n/a	n/a	n/a	n/a	n/a	n/a	n/a
Noodles in broth	Argentina	9	488	113	330	385	550	570	625
	Costa Rica	13	411	64	308	369	406	448	528
	Paraguay	23	322	80	156	273	306	367	507
	Peru	20	381	135	291	308	336	401	900
Wet and dry soups	Argentina	49	266	61	40	228	282	303	415
	Costa Rica	77	322	112	47	288	325	365	725
	Paraguay	22	290	30	223	282	288	305	351
	Peru	n/a	n/a	n/a	n/a	n/a	n/a	n/a	n/a

[1] Food categories as defined by Pan American Health Organization. SALT-SMART Consortium consensus statement to advance target harmonization by agreeing on regional targets for the salt/sodium content of key food categories. 2015; https://www.paho.org/hq/dmdocuments/2015/salt-smart-Consensus-statement-with-targets-FINAL.pdf.
[2] Sodium content was recorded "as consumed".

Commented [M2]: There is no explanation for "2" in the table.
